# Intelligent Fault Diagnosis of Hydraulic Piston Pump Based on Wavelet Analysis and Improved AlexNet

**DOI:** 10.3390/s21020549

**Published:** 2021-01-14

**Authors:** Yong Zhu, Guangpeng Li, Rui Wang, Shengnan Tang, Hong Su, Kai Cao

**Affiliations:** 1 National Research Center of Pumps, Jiangsu University, Zhenjiang 212013, China; zhuyong@ujs.edu.cn (Y.Z.); 2221911023@stmail.ujs.edu.cn (R.W.); 2111811013@stmail.ujs.edu.cn (S.T.); 2222011046@stmail.ujs.edu.cn (H.S.); 2222011002@stmail.ujs.edu.cn (K.C.); 2 State Key Laboratory of Fluid Power and Mechatronic Systems, Zhejiang University, Hangzhou 310027, China; 3 Ningbo Academy of Product and Food Quality Inspection, Ningbo 315048, China

**Keywords:** axial piston pump, intelligent fault diagnosis, deep learning, wavelet time-frequency analysis, convolutional neural network

## Abstract

Hydraulic piston pump is the heart of hydraulic transmission system. On account of the limitations of traditional fault diagnosis in the dependence on expert experience knowledge and the extraction of fault features, it is of great meaning to explore the intelligent diagnosis methods of hydraulic piston pump. Motivated by deep learning theory, a novel intelligent fault diagnosis method for hydraulic piston pump is proposed via combining wavelet analysis with improved convolutional neural network (CNN). Compared with the classic AlexNet, the proposed method decreases the number of parameters and computational complexity by means of modifying the structure of network. The constructed model fully integrates the ability of wavelet analysis in feature extraction and the ability of CNN in deep learning. The proposed method is employed to extract the fault features from the measured vibration signals of the piston pump and realize the fault classification. The fault data are mainly from five different health states: central spring failure, sliding slipper wear, swash plate wear, loose slipper, and normal state, respectively. The results show that the proposed method can extract the characteristics of the vibration signals of the piston pump in multiple states, and effectively realize intelligent fault recognition. To further demonstrate the recognition property of the proposed model, different CNN models are used for comparisons, involving standard LeNet-5, improved 2D LeNet-5, and standard AlexNet. Compared with the models for contrastive analysis, the proposed method has the highest recognition accuracy, and the proposed model is more robust.

## 1. Introduction

The hydraulic piston pumps are the core power source of the hydraulic transmission system, which are the “heart” of the hydraulic system. The reliability of its work is the key to ensure the high precision, high speed, stable operation of many national defense equipment and industrial equipment. Once the piston pump breaks down, downtime will occur, and the entire production line maybe paralyzed. More severely, it could even cause a catastrophic accident [1,2]. However, hydraulic pumps often face rigorous operating conditions such as high temperature, heavy load, high speed, and high pressure, which accelerate the deterioration of the health condition of the hydraulic pumps [3,4]. Therefore, the investigation on the intelligent fault diagnosis of the piston pump plays a practical and significant role in safe and efficient production, personnel health, and so on [5,6].

In recent years, due to the reliance of traditional mechanical fault diagnosis on expert experience and knowledge, the diagnosis process consumes a lot of human resources, which is gradually unable to meet the needs of industrial production. Encouragingly, the rapid development of artificial intelligence has profoundly changed human social life and promoted the intelligentialize of traditional industries. Correspondingly, intelligent diagnosis methods have gradually become mainstream. On account of the excellent data processing capabilities, many methods based on artificial intelligence have gradually been employed in the territory of mechanical fault diagnosis, such as convolutional neural networks (CNN) [7,8,9], autoencoder [9,10], deep belief networks [11,12], and recurrent neural networks [13,14].

Aiming at the difficult operation and maintenance of complex engineering system for health diagnosis, Tamilselvan et al. applied deep learning to accomplish mechanical fault diagnosis [15]. A multi-sensor health diagnosis method was proposed on the basis of deep belief network, which was considered to be the landmark breakthrough for fault diagnosis combining deep learning model [15]. Moreover, Jiang et al. combined multi-sensor information fusion with support vector machine (SVM) to realize the fault diagnosis of gear and rolling bearing [16]. Azamfar et al. stacked different frequency domains data into two-dimensional matrix as the input of CNN to implement fault diagnosis of gearboxes, and the effect is more accurate than traditional machine learning methods [17]. Luwei et al. fused vibration data of different positions and combined with artificial neural network (ANN) to realize fault diagnosis of rotating machinery [18]. On the basis of coherent composite spectrum (CCS), Yunusa-Kaltungo et al. realized fault diagnosis of rotating machinery via combining multi-sensor data with ANN [19]. Liu et al. utilized cascade-correlation neural network to realize fault diagnosis of mechanical equipment, which indicates that the result of multi-data fusion is better than single data [20]. Wang et al. alleviated the conflict between multi-sensors data via improving the sensor, which has good application in the field of fault diagnosis [21]. Based on Elman ANN, Kolanowski et al. built a navigation system, which can easily add other sensors and make data better fusion [22]. In addition, intelligent fault diagnosis generally includes two portions, feature extraction and self-learning classification of neural network model respectively [23]. For the sake of surmounting the problem of over-fitting created by the small amount of data in hydraulic pumps, Kim et al. combined with deep learning models to achieve the status detection of hydraulic system. It was worth pointing out that sensors were used to collect signals and meanwhile joggling was performed to simulate additional noise to expand the amount of sample data [24]. Zhang et al. used continuous wavelet transform (CWT) to obtain single-channel time-frequency diagrams of bearing vibration signals and merged three single-channel samples into three-channel samples as input data. The fault diagnosis was realized by using multi-channel sample data and demonstrated to be better than that of single-channel sample data [25]. Quinde et al. combined Wigner–Ville distribution with local mean decomposition (LMD) to realize bearing fault diagnosis based on one-dimensional signals [26]. Zhao et al. proposed a normalized CNN that combined batch normalization (BN) with exponential moving average (EMA) technology to construct a fault diagnosis model. The established model can be suitable for data imbalance and changing working conditions and obtained the desirable fault diagnosis performance for rotating machinery [27]. In terms of the structure of networks, Che et al. built a deep residual network model to tackle the problems such as complex fault types and long vibration signal sequences. The residual module was added to the CNN to further reduce the training error of the model, and intelligent fault diagnosis of bearing was finally achieved [28]. Aiming at the difficulties of feature extraction and poor robustness of the model, Wei et al. combined CNN with long short-term memory (LSTM) to achieve fault diagnosis of piston pumps with different cavitation degrees. The model still presented good robustness in the case of additional noise [29]. Kumar et al. introduced a new divergence function into the cost function, thereby reducing the complexity of the hidden layers, and finally the accuracy of diagnosis of centrifugal pump component defects was raised by 3.2% [30]. Al-Tubi et al. used genetic algorithms to adjust hidden layers of support vector machines to achieve fault diagnosis of centrifugal pumps [31]. Siano et al. combined fast Fourier transform with an artificial neural network to achieve the online detection of pump cavitation [32]. For the fault diagnosis of the piston pump, Du et al. built an integrated model and obtained the higher accuracy than the models for contrastive analysis. The model combined the sensitivity analysis (SA) of the characteristic parameters with the empirical mode decomposition (EMD). The higher sensitivity characteristics were input into probabilistic neural networks (PNN) for feature learning. The model still had good generalization performance in the multi-mode recognition state [33]. Wang et al. used a band-pass filter to improve the performance of minimum deconvolution and effectively detect the bearing failure of the piston pump [34]. Lu et al. used sparse empirical wavelet transform to process vibration signals of gear pump, combined with adaptive dynamic least squares support vector machine method (LSSVM) to achieve gear pump fault diagnosis, and the effect was better than empirical wavelet transform combined with LSSVM [35]. Although the above studies have adopted deep learning models in mechanical fault diagnosis and have achieved many beneficial research results, however, they require high signal processing-related knowledge in feature extraction and consume vast range of human resources in data processing. More importantly, deep learning is rarely utilized in the fault diagnosis field of hydraulic piston pump. The advantages of the deep learning models in feature self-learning need to be further explored.

The vibration signal of the hydraulic piston pump is a typical non-stationary signal [1,36]. The short-time Fourier transform, Wigner transform, and wavelet transform are widely utilized in the analysis of non-stationary signals [37,38,39]. Short-time Fourier transform has a defect of low resolution [40]. Wigner transform has so-called “cross-term” interference that cannot be explained and suppressed to multi-component signals [41]. The wavelet transform inherits the localization idea of the short-time Fourier transform and makes up for the weakness that the size of the sliding window does not change with frequency. It has high resolution and can well extract the time domain and frequency domain characteristics of non-stationary signals. Therefore, wavelet transform gradually becomes an important method to deal with non-stationary signals. The results of wavelet transform are displayed in the form of RGB images, which is essentially the response of the energy intensity of the signal at different times and frequencies. It can show the detailed changes of the signal and describe the fault characteristics in the signal [42]. Therefore, it can be used to extract the fault characteristics of the vibration signal of the piston pump, which provides an auxiliary path for the fault diagnosis of piston pumps.

This paper takes the hydraulic axial piston pump as the research object, a main contribution is in the following:

The constructed deep CNN simplifies the structure of the classic AlexNet network model. The classic AlexNet with five convolutional layers is reduced to the model with three convolutional layers. The full connected layer, convolutional layer and maxpooling layer are redesigned. The number of maxpooling kernel, convolutional kernel, and full connected layer neurons are adjusted. The LRN layer is removed on account of the minor influence on the diagnostic accuracy. The constructed deep CNN is trained based on dataset of real axial piston pump. Four optimizers are utilized in the gradient descent process of constructed CNN model, and the Adam optimizer is finally selected, which can make the model training process converge fastest, steady and improve generalization ability. Moreover, the hyperparameters are optimized for the enhance performance, including learning rate, batch size, the number and the size of convolutional kernel, and dropout rate. The quantity of convolution kernels are unified in each layer, and the quantity of nodes in the fully connected layer are improved. The RELU activation function is employed. The input data are three-channel feature images. The constructed deep CNN model is composed of three convolutional layers, three pooling layers, and three fully connected layers. Each pooling layer is connected behind each convolutional layer. The random inactivation neuron operation is added to the former two fully connected layers to avoid the overfitting of proposed model. The last layer is the softmax classifier for image classification. Compared with the classic AlexNet model, the structure is simplified, and the number of the parameters is enormously reduced in the improved CNN model. Moreover, the proposed model costs the shorter computation time and presents the higher classification performance compared with the other CNN models.

This article is composed as follows: in Section 2, the basic theory of CWT is briefly introduced. In Section 3, the principle of CNN is described, including the convolutional layer, pooling layer, and classification layer. In Section 4, the improvement of AlexNet model is described and analyzed. In Section 5, the proposed method is verified with measured fault data of hydraulic pump, and the test results are discussed. In Section 6, conclusions are drawn, and future research is prospected.

## 2. Continuous Wavelet Transform

Wavelet transform is extensive employed in the domain of mechanical fault diagnosis. CWT of signal can be expressed as follows [43,44,45]:

(1) $$\omega_{t}\left(\alpha ,\tau \right)=\left(f\left(t\right),\varphi_{\alpha ,\tau }\right)=\frac{1}{\sqrt{\alpha }}\int_{-\infty }^{\infty }f\left(t\right)\varphi^{*}\left(\frac{t-\tau }{\alpha }\right)dt$$

where $\varphi_{\alpha ,\tau }=\frac{1}{\sqrt{\alpha }}\varphi \left(\frac{t-\tau }{\alpha }\right)$ is the wavelet basis function, which is obtained from the mother wavelet function through a series of expansion and translation. α is the scale factor, which is related to frequency, if the value is larger, the corresponding time resolution is poor and the frequency resolution is good. τ is the shift factor, which is related to time. φ*(t) is the complex conjugate of φ(t).

The choice of wavelet basis is the most crucial step in wavelet transform [42]. In this paper, cmor wavelet was choosen as the wavelet basis function. After CWT, the one-dimensional signal f(t) is decomposed into wavelet coefficients related to the scale α and the shift τ, and then the two-dimensional time-frequency distribution images are projected.

## 3. Convolutional Neural Network

CNN is a deep learning method centered on image identification. It includes two parts, one is feature self-learning, and the other is classification. The network is composed of fully connected layers, pooling layers, convolutional layers, and so on. The feature self-learning is mainly operated in the convolutional layers. The classification task is performed in the softmax layer. To a certain degree, CNN benefits from the weight sharing mechanism of the convolutional layers and can reduce the number of training parameters. Now, more CNN structures with better generalization capabilities have been developed and applied in various fields, such as LeNet-5 [46], AlexNet [46,47], Vgg [48], and so on.

### 3.1. Convolutional Layer

As the core layer of CNN, most calculations are performed in the convolutional layer. It contains different feature information extracted by multiple convolution kernels [49]. Rich feature data can be extracted with deep convolutional layer. The convolution operation can be illustrated as the following equation:

(2) $$X_{j}^{L}=S\left(\sum_{i\in M_{j}}X_{i}^{L-1}\cdot w_{ij}^{L}+b_{j}\right)$$

where L is the current number of layer. $X_{i}^{L-1}$ is the input trait map of L−1 layer. $X_{j}^{L}$ is the output trait map of L layer. $w_{ij}^{L}$ is the weight matrix. $b_{j}$ is the bias value of convolution layer. $M_{j}$ is the input feature set. S(⋅) is the activation function.

After convolution layers, the Rectified Linear Unit (RELU) activation function is generally used for nonlinear transformation, which contributes to speed of gradient descent and avoids vanishing gradient. Its mathematical expression is as follows:

(3) $$g(x)=\max\left(0,X_{j}^{L}\right)$$

### 3.2. Pooling Layer

The pooling layer generally includes max-pooling, mean-pooling, and stochastic pooling layer. The pooling layers are employed to accomplish down-sample to input feature data [50]. The pooling operation can decrease the space size of the data and the quantity of parameters of each layer of the model. Moreover, the phenomenon of model overfitting can be effectively avoided. In this paper, the max-pooling layer is utilized to take the maximum value of the neural unit in the receptive field as the new feature value through the pooling kernel.

### 3.3. Softmax Classification

For multi-classification tasks, the softmax function is usually utilized in the end of the network to map the output value to the interval (0, 1). After processed by the softmax function, the output vector will be converted into the form of the probability distribution. Its mathematical expression can be expressed as follows [51,52]:

(4) $$J^{\left(i\right)}={\left[z^{1},z^{2},\cdots ,z^{m-1},z^{m}\right]}^{T}$$

(5) $$P_{k}=Soft\;\max\left(J^{\left(i\right)}\right)=\frac{e^{zk}}{\sum_{1}^{m}e^{zm}}$$

(6) $$P=Soft\;\max\left(J^{\left(i\right)}\right)=\left[\frac{e^{z_{1}}}{\sum_{1}^{m}e^{z_{m}}},\frac{e^{z_{2}}}{\sum_{1}^{m}e^{z_{m}}},\cdots \frac{e^{z_{m-1}}}{\sum_{1}^{m}e^{z_{m}}},\frac{e^{z_{m}}}{\sum_{1}^{m}e^{z_{m}}}\right]$$

where $J^{\left(i\right)}$ is the output vector of the output layer. $z^{1},\cdots ,z^{m}$ are the element values of the output vector of m category. $P_{k}$ is the probability value of input sample, which belongs to the *k*_th_ category. *P* is the probability distribution of the m category. $\frac{e^{zk}}{\sum_{1}^{m}e^{zm}}$ is the normalization function.

## 4. Intelligent Diagnosis Method Combining Wavelet Time-Frequency Analysis with Improved AlexNet Model

### 4.1. Improvement of AlexNet Network Model

The standard AlexNet model is a deep CNN, including 5 convolutional layers, 2 local response normalization (LRN) layers, 3 max-pooling layers, and 3 fully connected layers. It was born to solve image classification of 1000 types [53]. Compared with 1000 types of image recognition, signal classification of five working condition for the piston pump involved in this article is not considered to be a large-scale image recognition classification.

**Figure 1:**
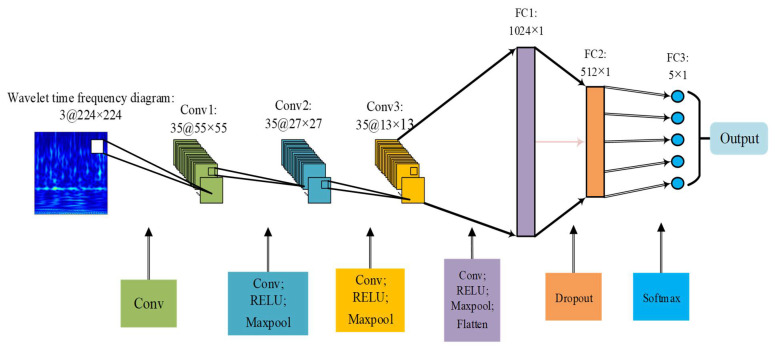
Improved AlexNet network model.

Considering the depth of the classic AlexNet network model, a large number of learning parameters and request for multiple GPUs to work at the same time, it makes model training more difficult. Thus, this paper simplifies the classic AlexNet model, unifies the quantity of convolution kernels in each layer, and improves the quantity of convolutional layers, the quantity of nodes in the fully connected layer, dropout value, and so on. We make use of the RELU activation function. The input data are 3-channel feature images. The model is composed of 3 convolutional layers, 3 pooling layers and 3 fully connected layers. Each pooling layer is connected behind each convolutional layer. The random inactivation neuron operation is added to the former two fully connected layers to avoid the overfitting of proposed model. The last layer is the softmax classifier for image classification. The structure of the model is shown in Figure 1.

### 4.2. Network Model Training Process

**Figure 2:**
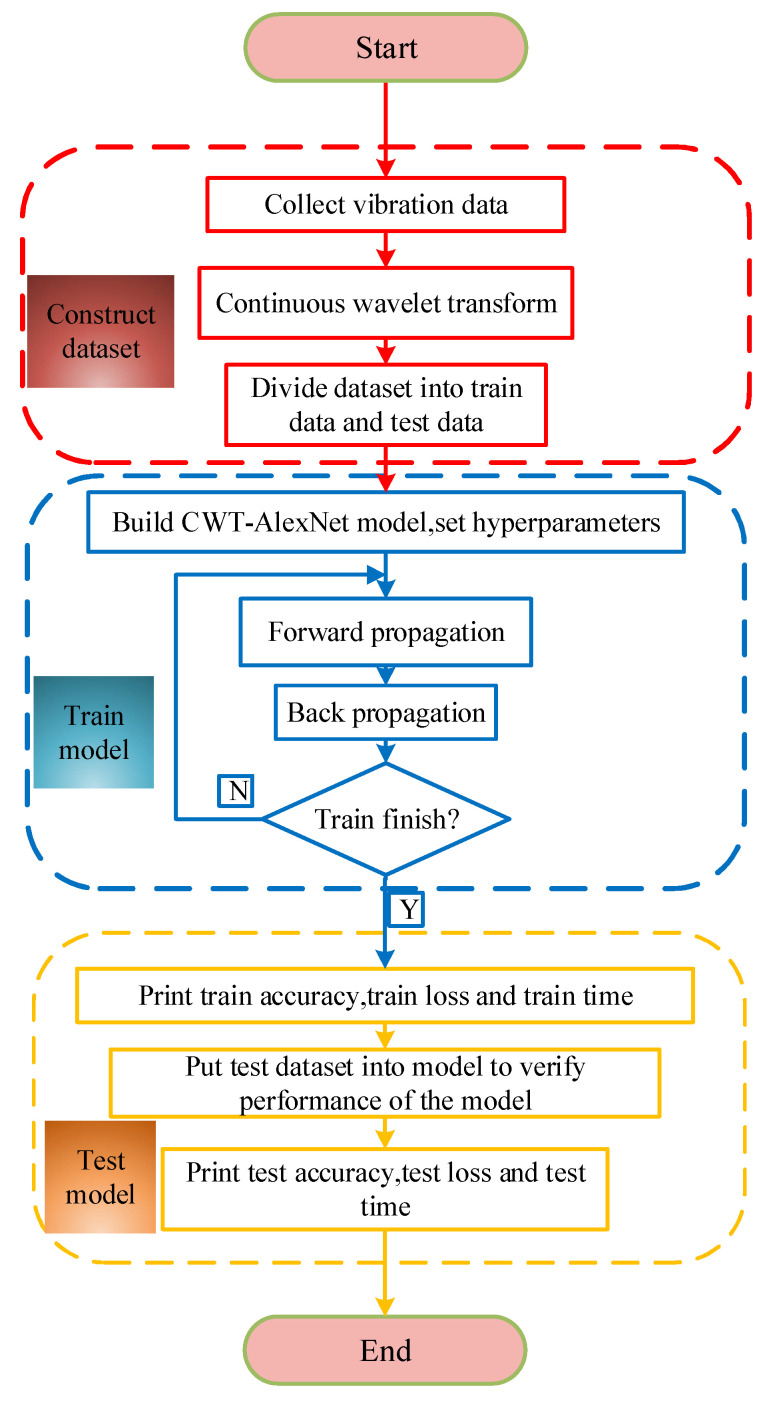
Network training process.

The size of time-frequency images is fixed as 224 × 224 through the resize function in Pytorch. Figure 2 reveals the flowchart of proposed model training. Firstly, the datasets are constructed and divided, and the mini-batch samples are taken as input to train model. Then, weight value, bias value, and other parameters are randomly initialized in the process of model training. During the model training, time-frequency graphs pass through the convolutional, pooling, fully connected layers, and feature data are forward propagated. The error value between the predicted output and the expected output is computed by cross-entropy cost function. In the meantime, weight value and bias value of each layer of the network are updated via back propagation. Finally, the training of the network is terminated with the purpose of reaching the convergence condition.

### 4.3. Process of the Intelligent Fault Diagnosis Method

The research ideas of the intelligent fault diagnosis method of piston pump on account of wavelet time-frequency analysis and improved AlexNet network model are as follows:

(1) The signal dataset is constructed by collecting the vibration signals of the piston pump test bench under different conditions. Then samples are constructed through the sliding window, and the length of each sample is 1024.
(2) Wavelet transform on the divided vibration signal dataset is performed to achieve the time-frequency distribution of one-dimensional time series, and 3-channel time-frequency images are generated. The division of dataset is in the following: the training sets account for 70% and the test sets account for 30%.
(3) The structural parameters of the diagnostic model are preliminarily set, such as learning rate, dropout value, the number of convolution kernel, and so on. Then a CNN structure based on the improved AlexNet model is established.
(4) The training loss and test accuracy of the model are taken as evaluation indicator to select structural parameters through numerous experiments.
(5) Through the above steps, the structural parameters of the neural network model are determined. Then, the training samples, test samples are input into the network model to retrain and verify the learning effect of the model, and t-distributed stochastic neighbor embedding (t-SNE) is utilized to visualize the effect of feature extraction.
(6) To further validate the diagnosis property of proposed model, the following deep models are used for comparisons, involving classic, improved 2D LeNet-5, classic LeNet-5, and classic AlexNet.

## 5. Experimental Verification

### 5.1. Sample Set

**Figure 3:**
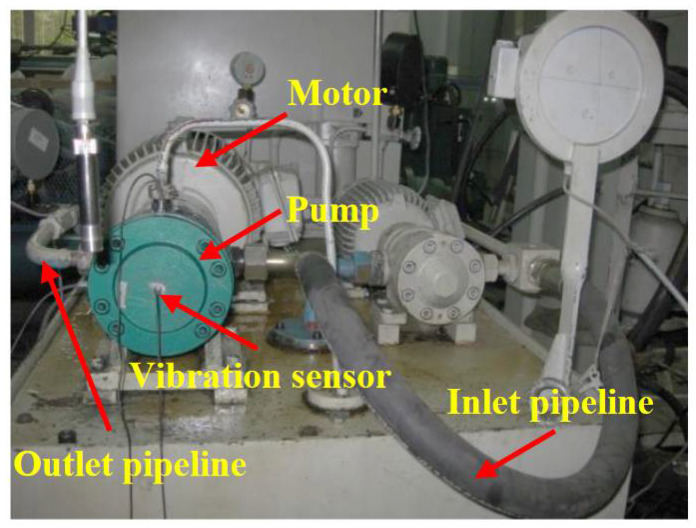
Hydraulic pump test bench.

For the sake of validating the effectiveness of the proposed method, a test bench is built to collect the vibration signals of the hydraulic pump in different working conditions. The experimental data collection was completed in Yanshan University. The test bench is shown in Figure 3. In the experiment, a swash plate axial piston pump is selected as the test object, and the type is MCY14-1B. The rated speed of motor is 1470 r/min, and it means the corresponding rotation frequency is 24.5 Hz. In the test, an acceleration sensor is installed at the end cover center of the pump to acquire the vibration signals, and the type is YD72D. The sampling frequency is 10 kHz.

**Figure 4:**
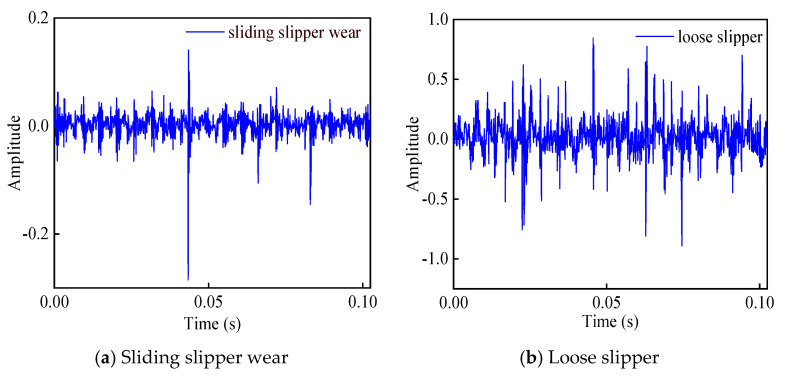

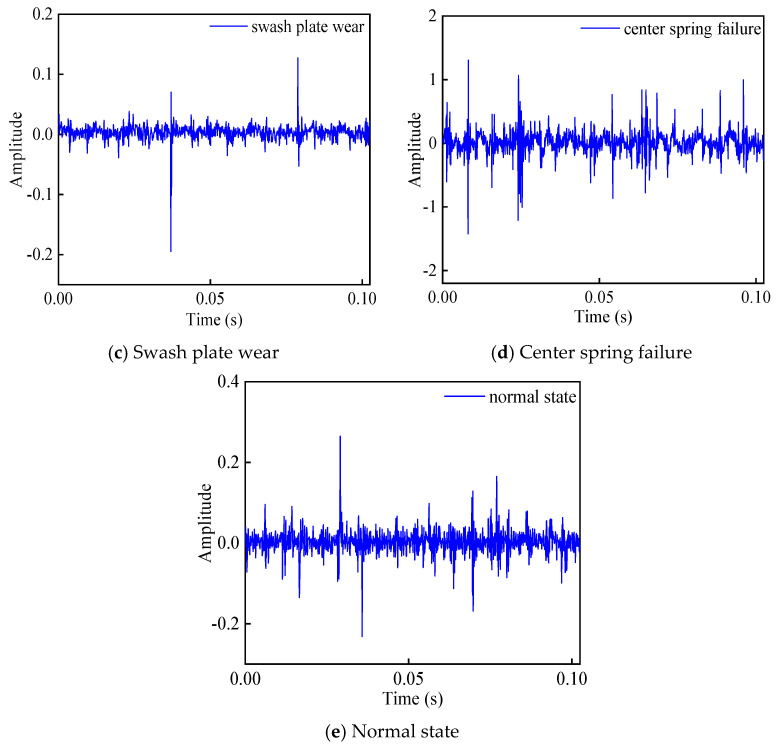
Time-domain waveform of five states.

During the experiment, the working pressure of the piston pump is respectively adjusted to 2 MPa, 5 MPa, 8 MPa, 10 MPa, and 15 MPa. Under each working pressure, the acceleration sensor is utilized to collect vibration signals of the piston pump in five states: normal state, sliding slipper wear, central spring failure, swash plate wear, and loose slipper. Among them, the selected four failure states are the common failure cases of piston pump. The partial time-domain waveforms of vibration signals are shown in Figure 4.

**Figure 5:**
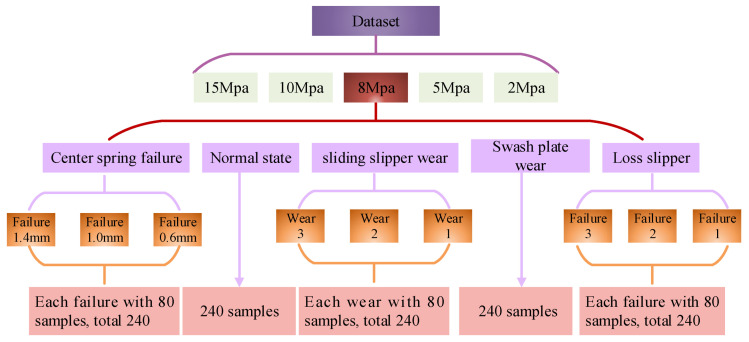
The composition of the sample set when the working pressure is 8 MPa.

In addition, in order to further validating the identification effect of the proposed method on different fault levels, three failure types with different degrees are set under the states of center spring failure, sliding slipper wear, and loose slipper. Three failure levels correspond to minor failures, moderate failures, and severe failures. Therefore, these malfunction data are respectively composed of three different failure sample sets. The composition of the sample set in five states at the working pressure of 8 MPa is listed in Figure 5. The composition of the sample set in other working pressure is the same as that of 8 MPa, including 2 MPa, 5 MPa, 10 MPa, and 15 MPa.

**Figure 6:**
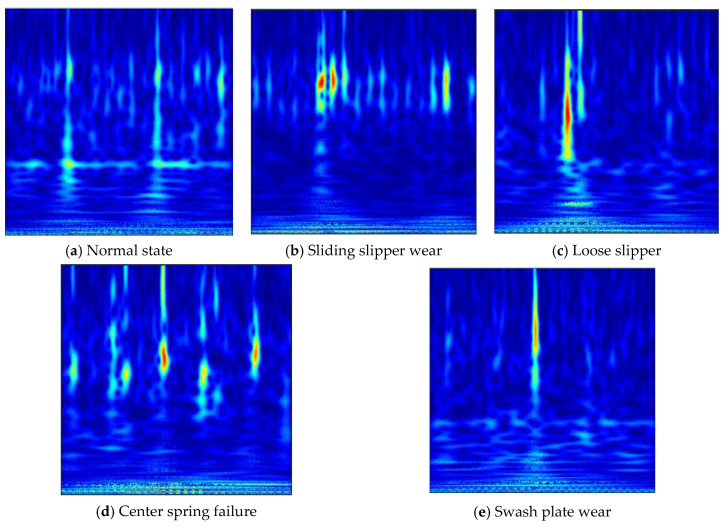
Time-frequency picture of five states of piston pump.

Seen from Figure 4, it is difficult to estimate the health status of the piston pump corresponding to the vibration signal via simply observing the time-domain waveform diagrams. Therefore, the vibration time-domain signal is converted into the time-frequency domain distribution with the wavelet time-frequency analysis method to highlight the internal characteristics. The partial wavelet time-frequency diagrams of five states of the piston pump are shown in Figure 6.

About the experiment, the wavelet time-frequency diagrams of the vibration signal are taken as the analysis sample for fault identification. Under each working pressure, the number of each state data sample is 240 and it means the total sample is 6000. The samples are arranged randomly. The composition of the sample-set is displayed in Table 1.

### 5.2. Optimal Selection of Model Structure Parameters

The selection of structure parameters plays a significant role in the construction of neural networks. The measured data analysis in this article is based on the deep learning framework PyTorch 1.5.1 and python programming language. The computer configuration is W-2235CPU @3.80 GHz, the graphics card is RTX4000, and RAM (random access memory) is 32 GB. The PyTorch framework is employed to initially build a CNN model, including 3 diverse convolutional layers, 3 uniform max-pooling layers, and 3 diverse fully connected layers. The batch size, learning rate, dropout value, and the number of convolution kernels are determined via debugging the parameter of the model. On behalf of ensuring the robustness of the experiment results, all experiments are repeated 10 times. The computational formula of the test accuracy is as follows:

(7) $$Model\;accuracy=\frac{n_{correct}}{N_{all}}\times 100\%$$

where $n_{correct}$ is the quantity of samples whose predicted label is consistent with the target label through the convolutional neural network. $N_{all}$ is the total quantity of samples in the training sample set or test sample set, respectively correspond to the training accuracy rate and test accuracy rate of the proposed model.

**Figure 7:**
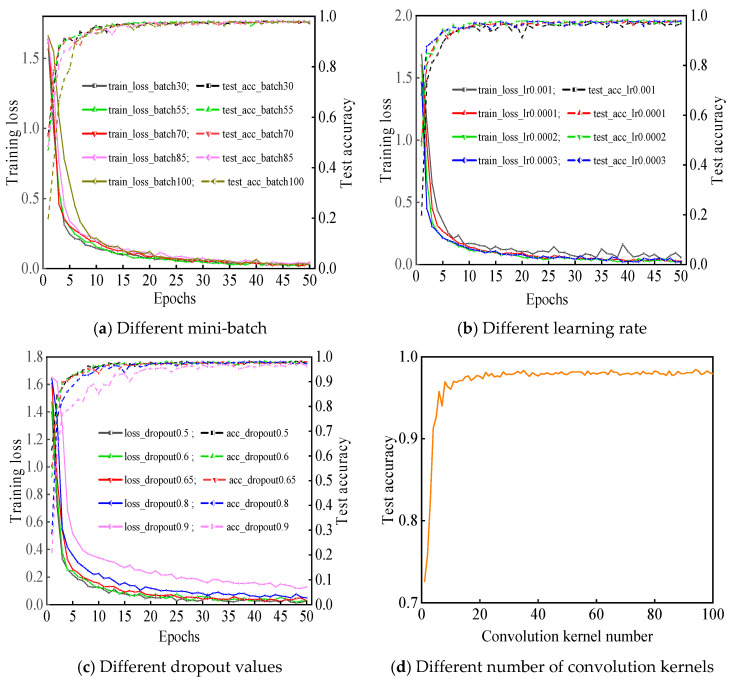

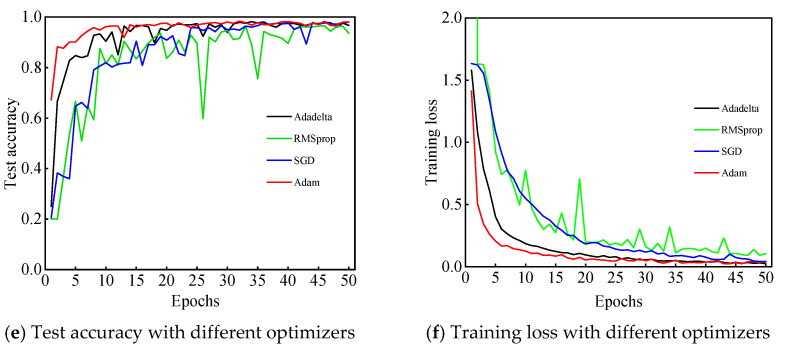
The tendency of different model parameters.

The consequences of debugging the model are revealed in Figure 7.

Seen from Figure 7a, with different batchsizes, the loss curves present different convergence rates. When the number of batchsize is 55, the loss curve converges faster and the training accuracy curve achieves stable in fewer epochs. To sum up, the overall effect is better than the other four batchsize.

In terms of different learning rates, Figure 7b reflects the changes of test accuracy and training error loss. When the learning rate is 0.001, the training error loss curve and test accuracy curve are undulating. The convergence effect of training error curve with learning rate of 0.0001 is poorer than that with learning rate of 0.0002 and 0.0003. When the learning rate is 0.0002 and 0.0003, the convergence speed of the error loss curve of the two training sets is small, but the convergence trend of the test set accuracy curve is more stable at the learning rate of 0.0002.

Seen from Figure 7c, different dropout values have different impact on the performance of the model with the same number of epochs. From the perspective of the training loss curve, when the dropout value is 0.8 and 0.9, the convergence speed of loss curve is slower, and the test accuracy curve of the model displays great fluctuation. It can be indicated that the larger dropout value leads to the insufficient feature extraction and the unfavorable learning effect. However, the average error of the model is small, and the convergence speed of error loss curve is fast at the dropout value of 0.5. At the same time, the test accuracy curve converges rapidly and presents a steady trend. Moreover, a higher convergence accuracy is obtained.

The learning effect under different numbers of convolution kernels are displayed in Figure 7d. It can be seen that the learning effect is not good, and the test accuracy is low within the interval (1, 20). With the increase of the number of convolution kernels, the eigenvalues extracted by the model are more representative. In the meantime, the test accuracy of proposed model gradually augments and tends to be stabilized. The test accuracy value is close to the maximum value at the number of convolution kernels of 35. As the number of convolution kernels is directly proportional to the computational complexity of the model, it already meets the needs of sample testing at the quantity of convolution kernels in each convolution layer of 35.

The selection of appropriate optimizer can make the training loss of the model decrease quickly and steadily. Seen from Figure 7e,f, the accuracy curve of the model fluctuates sharply with the RMSprop optimizer. When Adadelta, SGD, and RMSprop optimizers are employed in the model, the initial accuracy of the model is all low. With the epochs increasing, although the accuracy gradually increases, the accuracy of the model fluctuates greatly. Nevertheless, the higher accuracy and lower training loss value are attained in the initial training stage when Adam optimizer is employed. The corresponding accuracy curve converges faster, and the training loss curve falls smoother than that with Adadelta, SGD, and RMSprop. When the epoch reaches 15, the optimal accuracy is achieved.

According to the above analysis, the structure of the proposed model is elected as follows: the batchsize is 55, the learning rate is 0.0002, the dropout value is 0.5, the quantity of convolution kernels is 35, and the optimizer is Adam. The structure parameters of each layer of the model are revealed in Table 2.

### 5.3. Fault Diagnosis Based on CWT-AlexNet

**Figure 8:**
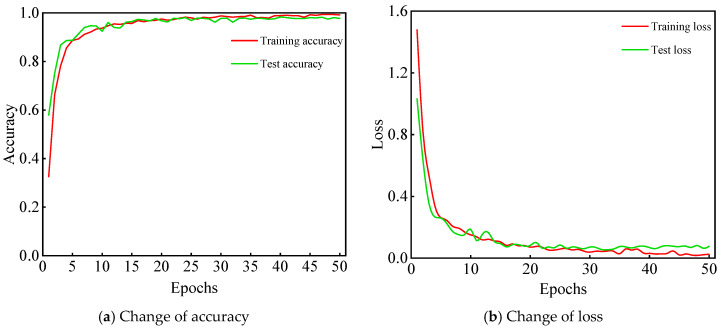
The curve of accuracy and loss error.

Based on randomly initialized weight value and bias value, a fault diagnosis model is built. The cross-entropy loss function is employed to calculate the loss value between output labels and real labels, and an Adam optimization algorithm is utilized to update the weight value and bias value of each layer. The training of model is not terminated until the training loss curve and test accuracy curve no longer decline or rise greatly with the increase of epoch. After repeating experiments 10 times, the average accuracy of the model is 98.06%. The highest accuracy can reach 98.33%. The accuracy curves and loss curves of the model are shown in Figure 8.

The recognition accuracy of each state test sample based on proposed model is revealed in Table 3.

Seen from Table 3, the features of the vibration signals of the piston pump can be extracted via the fault diagnosis model with CWT-AlexNet, and the different types of faults are distinguished effectively. Among the signals of the piston pump, the recognition accuracy of the normal state, sliding slipper wear, and swashplate wear all achieve 100%, which indicates that the hidden characteristics of the vibration signals of these three states can be self-learned by the diagnostic model. Due to the similarity of the characteristics of the vibration signals of loose slipper failure and center spring failure, it may easily cause misclassification. Therefore, the corresponding recognition accuracies respectively only reach 97.22% and 94.72%.

**Figure 9:**
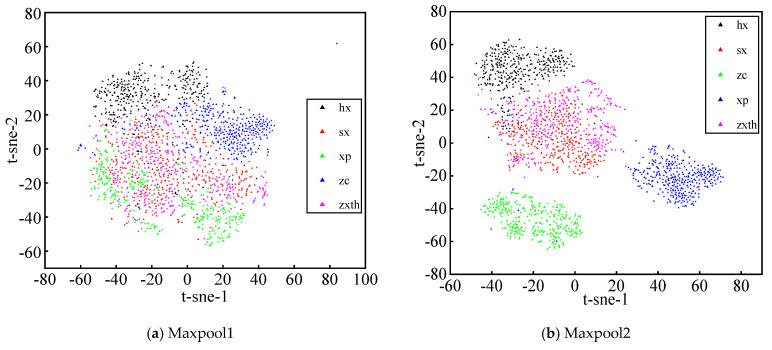

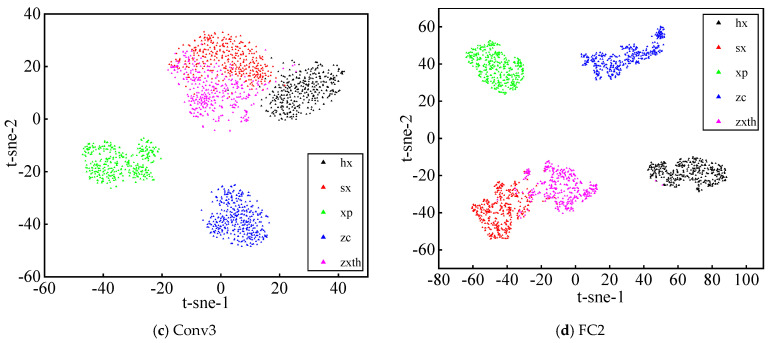
Visualization of t-distributed stochastic neighbor embedding (t-SNE). where, hx is the sliding slipper wear, sx is the loose slipper, xp is the swash plate fault, zc is the normal state, and zxth is the center spring failure.

In order to further clearly show the feature extraction and classification capabilities of the model, t-SNE is utilized to visualize the process of feature extraction of some middle layers. Seen from Figure 9, the visual clustering effects of the followings are analyzed, including the first max-pooling layer (Maxpool1), second max-pooling layer (Maxpool2), third convolution layer (Conv3), and penultimate fully connected layer (FC2). Through the feature extraction of the Maxpool1 layer, it can be seen that the feature data of the piston pump are mixed with each other, and difficult to distinguish. However, after extracted by the FC2 layer, the input features represent good five cluster distribution. It can be observed that the same fault signatures congregate with each other and the different fault signatures repel each other, which indicates that the model has good classification and recognition ability. It means the ability of feature extraction of the model is gradually enhanced with the deepening of the neural network.

### 5.4. Comparative Verification

In order to validate the feature extraction availability of the proposed model, the performance of the CWT-AlexNet network is compared with other commonly used models, including standard LeNet-5, AlexNet and improved 2D LeNet-5 [54] network. The detailed setting of improved 2D LeNet-5 network can be searched in [54]. After repeating experiments 10 times, the average test accuracy, standard deviation (Std), training time, and test time are taken as evaluation indicators. Comparison consequences are revealed in Table 4.

Seen from Table 4, the average accuracy of the four models is all above 90%. The CWT-AlexNet model has obvious advantages in comparison with traditional LetNet-5 and improved 2D LeNet-5 model. The average accuracy of the CWT-AlexNet model is respectively higher than LetNet-5 and improved 2D LeNet-5 model about 4.27% and 2.43%, and the Std of the proposed model is lower. When compared with the classic AlexNet model, the average accuracy of the CWT-AlexNet model increases only 0.4%; however, the model takes less calculation time, and the diagnostic efficiency is better than the classic AlexNet model.

**Figure 10:**
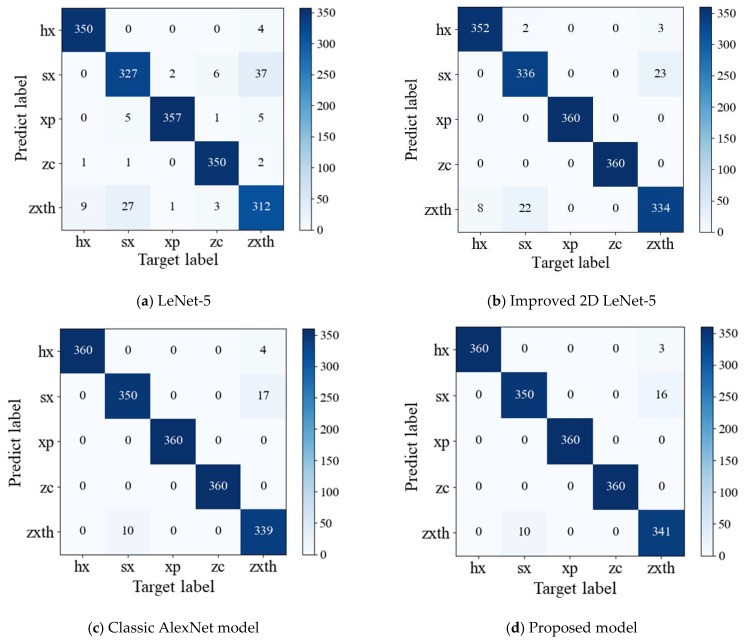
Comparison of model classification effect.

For the purpose of visually show the performance of the model for multi-fault classification prediction, the classification effect confusion matrix of the above four models are shown in Figure 10.

Figure 10a–d are the confusion matrix of LeNet-5, improved 2D LeNet-5, classic AlexNet and CWT-AlexNet model, which reflect the misclassification of the five state signal samples. According to the results presented by the confusion matrix, four models have good performance on the signals under normal state, sliding slipper wear, and swashplate wear, and the number of misclassified samples is small. The proposed model performs best on center spring failure samples and loose slipper failure, and the quantity of misclassifications is less than that of the classic AlexNet model, LeNet-5 and improved 2D LeNet-5 models.

**Figure 11:**
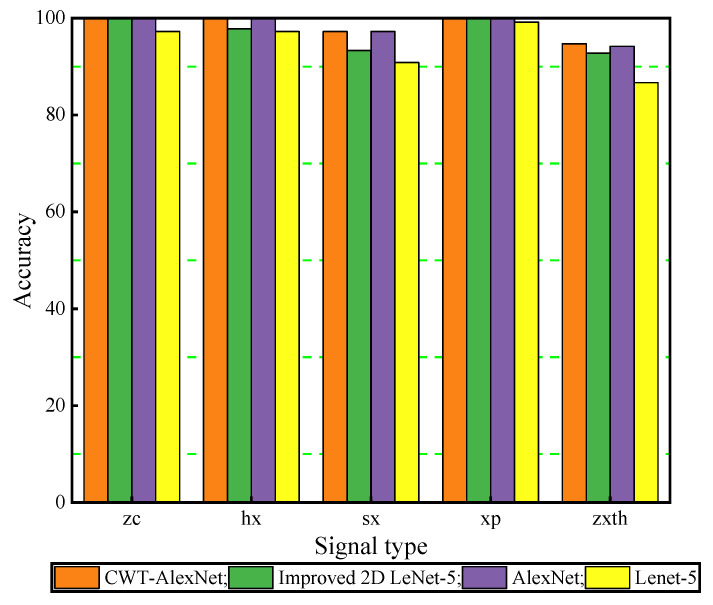
Histogram of the classification results for different state signals.

For the purpose of intuitively compare the correct classification results of above models. The histograms of the signal classification in different states are shown in Figure 11. It vividly shows that the CWT-AlexNet model has the highest recognition accuracy for the five state signals, which further illustrates that the diagnostic model has higher recognition accuracy and stronger model robustness.

## 6. Conclusions

In this study, a novel intelligent fault diagnosis method is proposed via combining CWT with CNN, which fully integrates the ability of wavelet time-frequency analysis in feature extraction and the ability of AlexNet in automatic learning.

(1) The structure of AlexNet network is improved through reducing the number of parameters and calculation complexity of each layer. The proposed model can extract features from the vibration signals of the piston pump in different states and identify various fault types effectively. The recognition accuracy of the normal state, sliding slipper wear, and wear swash plate fault can reach 100%, the recognition accuracy of the loose slipper fault can reach 97.22%, and the recognition accuracy of the center spring failure can reach 94.72%.
(2) Compared with standard LeNet-5 network, standard AlexNet network and improved 2D LeNet-5 network, the proposed CWT-AlexNet model has the highest recognition accuracy for five fault types of the piston pump, and the proposed model has strong robustness.

This research will provide a theoretical reference for the intelligent fault diagnosis of piston pump and conducive to the failure prediction of piston pump.

## Figures and Tables

**Table 1:** Signal samples and labels.

Sample Type	Total	Training Sample	Test Sample	Labels
Sliding slipper wear	1200	840	360	0
Loose slipper	1200	840	360	1
Swashplate wear	1200	840	360	2
Normal state	1200	840	360	3
Center spring failure	1200	840	360	4
Total	6000	4200	1800	—

**Table 2:** Parameters of each layer.

Layers	Convolution Kernels × Convolution Kernel Size	Output	Activation Function
First convolution layer	35 × 11 × 11	35 × 55 × 55	RELU
First max-pooling layer	35 × 3 × 3	35 × 27 × 27	—
Second convolution layer	35 × 3 × 3	35 × 27 × 27	RELU
Second max-pooling layer	35 × 5 × 5	35 × 13 × 13	—
Third convolution layer	35 × 3 × 3	35 × 13 × 13	RELU
Third max-pooling layer	35 × 3 × 3	35 × 6 × 6	—
First full connection layer	—	1024	RELU
Second full connection layer	—	512	RELU
Third full connection layer	—	5	—

**Table 3:** The recognition accuracy rate of each state.

Sample Type	Recognition Accuracy
Normal state	100.00%
Loose slipper	97.22%
Sliding slipper wear	100.00%
Swash plate fault	100.00%
Center spring failure	94.72%

**Table 4:** Comparison with different models.

Model	Average Accuracy	Std	Training Time (s/epoch)	Test Time (s/epoch)
CWT-AlexNet	98.06%	0.171	7.27	3.12
Tradition LeNet-5	93.79%	0.348	5.33	1.41
Improved 2D LeNet-5	95.63%	0.739	10.29	1.53
Tradition AlexNet	98.02%	0.134	11.54	4.25

## Data Availability

The data presented in this study are available on request from the corresponding author upon reasonable request.
